# Connections Between Febrile Infection-Related Epilepsy Syndrome and Autoimmune Encephalitis. A Case Report of a Child With New Anti-neuronal Antibodies

**DOI:** 10.3389/fped.2022.908518

**Published:** 2022-08-08

**Authors:** Martina Basso, Matteo Gastaldi, Valeria Leonardi, Giana Izzo, Sara Olivotto, Stefania Ferrario, Pierangelo Veggiotti, Diego Franciotta, Stefania M. Bova

**Affiliations:** ^1^Department of Biomedical Sciences and Clinics Luigi Sacco, Faculty of Medicine and Surgery, University of Milan, Milan, Italy; ^2^Neuroimmunology Laboratory, IRCCS Mondino Foundation, Pavia, Italy; ^3^Department of Pediatric Radiology and Neuroradiology, V. Buzzi Children’s Hospital, ASST Fatebenefratelli Sacco, Milan, Italy; ^4^Pediatric Neurology Unit, V. Buzzi Children’s Hospital, ASST Fatebenefratelli Sacco, Milan, Italy; ^5^Department of Pediatrics, Division of Anesthesia and Intensive Care, V. Buzzi Children’s Hospital, ASST Fatebenefratelli Sacco, Milan, Italy; ^6^Neuroimmunology Laboratory, IRCCS Ospedale Policlinico San Martino, Genoa, Italy

**Keywords:** FIRES (febrile infection-related epilepsy), autoimmune encephalitis (AE), anti-neuronal antibodies, claustrum abnormalities, plasma exchange (plasmapheresis), case report

## Abstract

Acute encephalitis and febrile infection-related epilepsy syndrome (FIRES) are debilitating neurological disorders. It is increasingly accepted that FIRES should be considered an autoinflammation-mediated epileptic encephalopathy, but the debate about its etiopathogenesis is still very much open. Despite showing a considerable overlap with encephalitis, it continues to be regarded as a distinct entity. We describe the case of a previously healthy 5-year-old child who, following a fever, developed acute encephalopathy, status epilepticus, neurological, neuropsychological, and psychiatric manifestations, and claustrum involvement on MRI. At symptom onset, his clinical and instrumental data met the diagnostic criteria for both FIRES and acute encephalitis. He received benzodiazepines, levetiracetam, phenytoin, phenobarbital, thiopental, and first-line immunotherapy for acute inflammatory encephalopathy (intravenous methylprednisolone and immunoglobulins), without substantial improvement. Following the detection of anti-neuronal antibodies through immunohistochemistry performed on rat brain slices, he received therapeutic plasma exchange (TPE). His neurological and behavioral conditions improved drastically and his antibody titer fell sharply from the first to the last course of PE. Claustrum abnormalities on MRI disappeared. The patient’s long-term outcome is favorable. At 13 months after discharge, he experienced a focal seizure and carbamazepine was started, achieving seizure control. At 10 years of age, he is still on carbamazepine, with well-controlled seizures, focal EEG abnormalities, and an otherwise normal neurological and cognitive profile and normal MRI. This case strengthens the view that FIRES might constitute the initial clinical presentation of a CNS inflammatory disease that could have, among multiple distinct etiologies, an autoimmune cause. Immunological and specific second- or third-level investigations including immunohistochemistry should be included in the diagnostic work up of patients with FIRES-like phenotypes. PE could be effective in this subset of patients, protecting them from long-term neurological sequelae.

## Introduction

Acute encephalitis is a usually infectious debilitating neurological disorder. However, an increasing number of non-infectious, mostly autoimmune, cases are now coming to light. These new autoimmune forms may be associated with antibodies against neuronal cell-surface or synaptic proteins. Patients can present infectious encephalitis-like core symptoms or neurological and psychiatric manifestations without fever or cerebrospinal fluid (CSF) pleocytosis. Little has thus far been established with regard to the diagnostic work up and therapeutic management of these forms, which will likely show a broadening clinical spectrum.

Febrile infection-related epilepsy syndrome (FIRES) is a clinical condition characterized by *de novo* onset of refractory status epilepticus without any clearly identifiable acute or active structural, toxic, or metabolic cause in a previously healthy individual (1, 2). Its diagnosis is still based on clinical criteria. A febrile illness precedes seizure onset by 1–14 days. Seizure frequency increases, leading to refractory status epilepticus with hundreds of seizures per day. This phase is followed by a chronic phase with refractory epilepsy, and neurological and cognitive impairment (2). There are no biological markers or specific diagnostic tests (3). EEG recordings show recurrent extreme delta brush, and a distinctive seizure pattern consisting of focal activity > 10 Hz of small to moderate amplitude evolving into well-formed rhythmic spike and spike-wave complexes (4). A shifting or migratory seizure pattern is considered indicative of FIRES/new-onset refractory status epilepticus (2, 4). Bilateral claustrum hyperintensity and residual cortical atrophy have been observed on brain MRI (5).

Despite showing a considerable overlap with encephalitis, FIRES continues to be regarded as a distinct entity. Investigations into metabolic or genetic causes have been negative (6). Although there have been reports of overproduction of proinflammatory cytokines and of chemokines with proconvulsive activity, suggesting an underlying inflammatory mechanism, efforts to identify infectious causes have been unproductive: neuronal autoantibodies, such as serum anti-neuropil antibodies, VGKC-complex antibodies, GABA(A) receptor antibodies, anti-GAD antibodies, and anti-GluR3 antibodies, have been detected only in isolated cases (7–9). Recent studies on serum and CSF levels of IL1beta and IL1RA showed changes in the IL1 pathway, supporting the idea that FIRES is an autoinflammation-mediated epileptic encephalopathy (10–12).

To date, no specific therapy for FIRES is available (13). However, given the putative causal role of inflammation, various immune system-modifying treatments have been tried [i.e., steroids, intravenous immunoglobulins (IVIg), therapeutic plasma exchange (TPE), anakinra, tocilizumab, and ketogenic diet] (2), with mixed results.

## Case Report

**FIGURE 1:**
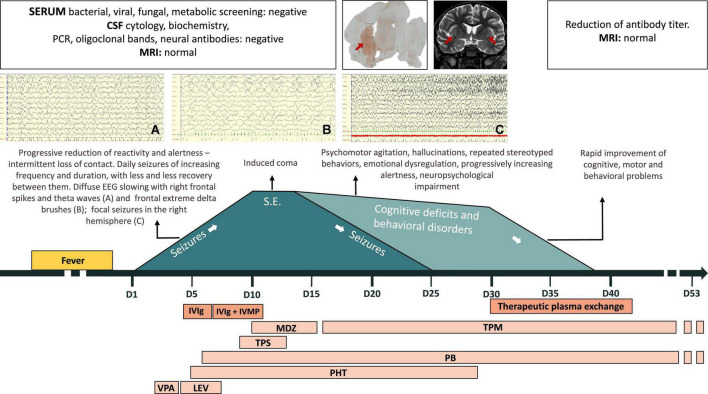
Patient’s clinical course. CSF, cerebrospinal fluid; IVIg, intravenous immunoglobulins; IVMP, intravenous methylprednisolone; LEV, levetiracetam; MDZ, midazolam; MRI, magnetic resonance imaging; PB, phenobarbital; PCR, polymerase chain reaction; PHT, phenytoin; S.E., status epilepticus; TPM, topiramate; TPS, thiopental; VPA, valproate.

Figure 1 summarizes the patient’s clinical course. Information enabling the patient to be identified has been removed. The patient is a previously healthy 5-year-old boy born to non-consanguineous healthy Caucasian parents. His family history is positive for autoimmune disease (celiac disease in his sister and autoimmune liver disease in a grandfather), but not for neurological disorders.

At the age of 5 years, he developed a fever after a gum infection, successfully treated with antibiotics.

The day after the fever resolved, he displayed severe somnolence and focal epileptic seizures with loss of contact and eye deviation (day 1). Over the following days, the seizures, lasting up to 20 min and characterized by loss of contact, eye deviation, and chewing/swallowing followed by clonic jerks of the left limbs, became increasingly frequent with progressive reduction of reactivity and alertness. EEG showed a pattern of very diffuse slowing, frontal extreme delta brushes, focal spikes and theta waves in the right frontal region, and seizures with focal fast activity in the right hemisphere. Brain MRI was normal. CSF physical and chemical analysis and oligoclonal bands were normal, as were polymerase chain reactions for herpes simplex virus (HSV), varicella zoster virus (VZV), enterovirus, and Epstein-Barr virus (EBV). Blood tests for bacterial, viral, or fungal infections and plasma antibodies, in particular for cytomegalovirus (CMV), Coxsackie virus, EBV, hepatitis B virus (HBV), hepatitis C virus (HCV), HIV1-2, HSV1-2, human herpes virus 6 (HHV6), mycoplasma pneumoniae, and parvovirus, were negative. Antiepileptic treatment (valproate up to 30 mg/kg and levetiracetam up to 50 mg/kg) and empirical antibiotic and antiviral treatments were started.

Due to progressive worsening of the patient’s mental status, he was moved to our intensive care unit on day 5, when EEG showed slowing, synchronization of abnormalities, and recurrent focal electrical and electro-clinical seizures in the right temporal area, refractory to phenytoin and phenobarbital. Brain MRI was still normal. Extensive work up for metabolic diseases was negative. Given the suspicion of immune-mediated encephalitis, immunomodulatory therapy with IVIg (2 g over 3 days) was added to his treatment. The first sample of CSF collected from the patient was tested for the presence of neuronal antibodies (NMDAR, LGI1, CASPR2, GABA-B, GluR, GluR2, anti-Hu, anti-Yo, anti-Ri, anti-Tr, anti-AMPH, anti-GAD, anti-Cv2, and anti-Ma); the findings were unremarkable.

On day 7, high-dose intravenous (IV) methylprednisolone (IVMP) was added (30 mg/kg/day for 5 days), to no effect.

On day 8, IV continuous infusion thiopental was started on day 10, a burst suppression pattern was achieved. Thereafter, midazolam was added from days 10 to 16, while thiopental was withdrawn by day 13. The EEG improved, although disorganization of electrical activity and bilateral epileptiform discharges persisted. MRI remained normal.

On day 16, sedative medication was discontinued and the child awoke. Over the following days, severe language and behavioral impairments emerged: eye contact was erratic and speech absent; he smiled and laughed frequently and inappropriately. He also presented generalized weakness, left hemiparesis, and severe insomnia. Brief focal seizures persisted (loss of contact, eye deviation, swallowing). Topiramate (10 mg/kg) was started and the seizures gradually abated, finally disappearing on day 25. Over the following days, speech remained absent, and hallucinations, as well as echopraxia, repeated stereotyped behaviors, and episodes of psychomotor agitation, appeared.

**FIGURE 2:**
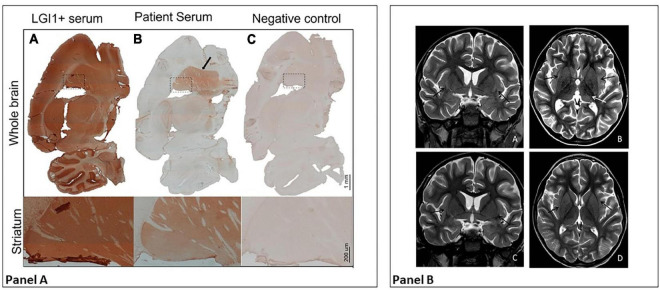
Panel **(A)** Diffuse staining is typical of certain anti-neuronal antibodies, such as anti-LGI 1 antibodies (A); in their presence, staining involves the hippocampus, cerebellum, thalamus, striatum (shown in detail), and cortical areas. The patient’s serum shows LGI1-like staining, but with selective involvement of the striatum (B, in detail). As shown in (C), no staining is observed with a control serum. Panel **(B)** MRI at day 40 (first row) vs. follow-up MRI 2 weeks later (second row). In the first row, axial and coronal T2-weighted images (A,B) show bilateral slight enlargement and hyperintensity of the claustrum (black arrows) and mild T2 hyperintensity of the left thalamic pulvinar (white arrow). Also note the diffuse widening of the pericerebral CSF spaces. After 2 weeks, axial and coronal T2-weighted images (C,D) show complete resolution of signal alterations and a quite normal appearance of the pericerebral CSF spaces.

Meanwhile, serum and CSF samples obtained before starting the immunomodulatory therapy were tested using immunohistochemistry on lightly fixed rat brain slices (14). Both serum and CSF showed intense neuropilar staining selectively involving the striatum (Figure 2A, serum titer: 1:1,200, CSF titer: 1:5), suggesting the presence of antibodies directed against an uncharacterized neuronal target. At this point, MRI showed slight bilateral enlargement and hyperintensity of the claustrum on T2-weighted sequences and mild T2 hyperintensity of the left thalamic pulvinar, without diffusion restriction or contrast enhancement after IV gadolinium administration (Figure 2B). Generalized widening of the pericerebral CSF spaces was also present, indicating reduced brain volume.

In the light of these results, on day 30 we decided to start TPE. The patient received 6 courses of PE from days 30 to 42. His neurological and behavioral conditions improved drastically: the hemiparesis disappeared; he resumed walking and eating independently, and behaving appropriately, understanding simple sentences and expressing himself verbally, initially through single words and subsequently simple sentences. His antibody titer fell sharply from the first to the last course of TPE. By day 42, claustrum abnormalities on MRI were no longer appreciable.

The child was finally discharged (day 53) in good general clinical conditions. His neurological examination was normal, while neuropsychological assessment showed moderate expressive aphasia and mild receptive aphasia with normal general cognitive abilities. Brain MRI was unremarkable, EEG recordings showed focal slowing and epileptiform discharges in the left temporal and right posterior regions. Phenobarbital and topiramate were continued until 4 and 10 months after discharge, respectively. At 13 months after discharge, by which time he had been seizure free for 1 year with a normal EEG and normal neurological and cognitive development, the patient experienced a focal seizure characterized by oral automatism and leftward eye deviation. EEG showed epileptiform discharges in the right posterior regions, while MRI was still normal and the antibody titer still low (1:200). Carbamazepine was started, achieving seizure control.

At the latest follow-up at 10 years of age, he is a healthy boy with a normal IQ. He is still on carbamazepine. Focal abnormalities persist on EEG; brain MRI and neurological examination are still normal. His antibody titer remains low (1:200).

Neither screening for preneoplastic conditions nor pediatric follow-up have ever suggested the presence of tumors.

## Discussion

We here described the case of a previously healthy child who, following a fever, developed acute encephalopathy, status epilepticus, neurological and psychiatric manifestations, and claustrum involvement. At symptom onset, his clinical and instrumental data met the diagnostic criteria for both FIRES and acute encephalitis; brain imaging findings were normal, as is usual in the initial phase. He received first-line immunotherapy for acute inflammatory encephalopathy (IVMP, IVIg), as well as several courses of TPE following the detection of anti-neuronal antibodies through immunohistochemical staining of rat brain slices, which showed selective involvement of the striatum. This finding could explain the appearance in the acute phase of signal abnormalities in the claustrum and, to a lesser extent, the thalamus; moreover, in a recent literature review on neuroimaging findings in FIRES (131 patients with a median age of 8 years), bilateral basal ganglia signal alterations were the second most frequently reported abnormalities in the acute phase (9/131), after peri-insular changes (claustrum included) (15). In the chronic phase of the disease, the authors reported evidence of brain atrophy in 48/95 patients submitted to neuroimaging follow-up. Temporal sclerosis was present in 24/95 patients, thus temporal lobe signal abnormalities (including hippocampal structures), frequently reported in the acute phase of FIRES, might be a result of prolonged seizures rather than manifestations of the encephalitis process itself. In our case, some brain atrophy was detected during hospitalization, which might even be attributed to antiepileptic drugs and cortisone therapy. At further follow-up, MRI was normal, maybe as a consequence of the prompt TPE.

Our patient responded well and rapidly to the treatment. His disease course was favorable. At 13 months after discharge, in the absence of clear triggers, he experienced a seizure and epileptiform discharges reappeared on EEG; carbamazepine was started, achieving seizure control. Unfortunately, the cause of this seizure recurrence is not easy to establish. There were no imaging abnormalities, and the antibody target was not definitively identified. Moreover, the patient’s serum titer has remained low over the years. Nevertheless, the evolution of the patient’s clinical picture over time has not been significantly compromised by this event, and indeed, several years on, he has a normal neurological and cognitive profile, and normal MRI, and focal epilepsy with well-controlled seizures.

The present report concerns the diagnostic and therapeutic management of acute encephalopathies and the question of the etiology of FIRES.

In this patient, uncharacterized neuronal antibodies were detected using immunohistochemistry on rat brain slices, a tissue-based assay that can detect most of the currently known neuronal surface antibodies, and thus allow broad screening of biological samples. The importance of this test and of anti-neuronal antibodies is still debated. Some authors have suggested that, to be considered relevant, their presence needs to be confirmed by testing on live neuronal cell cultures, which was not done in our patient (16). Staining patterns involving the striatum have been described with other neuronal antibodies, in particular anti-dopamine D2 receptor antibodies (17). These antibodies are usually found in children affected by autoimmune encephalitis with predominant movement disorders; seizures have been described in a minority of patients. However, cytometric bead array failed to detect D2 receptor antibodies in our patient, and the antibody target remains unknown. Nevertheless, the detection of neuronal antibodies provided the rationale for performing PE in our patient, and its effects were dramatic, supporting a role for circulating autoantibodies in causing and sustaining his complex neurological picture. Though the efficacy of immunomodulating treatments in FIRES is still debated, it is not unusual for similar patients to have received first-line and often more aggressive immune therapy. Our observation, as well as others in the literature, confirm that at least a subset of children with FIRES phenotype and neural antibodies are likely to respond to immune therapy (18).

In our patient, TPE proved more effective than IVIg, probably because it allowed direct and immediate removal of pathogenic autoantibodies (albeit unidentified in this case) and other disease mediators from the periphery, with immunomodulatory effects on the CNS. This observation, too, is confirmed in the literature. Indeed, there is evidence of improved outcomes in children diagnosed and treated early for several disorders including acute disseminated encephalomyelitis and anti-NMDAR encephalitis, both conditions in which PE plays an important role in first-line immunotherapy (19–21).

It has been hypothesized that repeated and prolonged seizures and exposure to a large number of drugs during the acute disease phase may damage frontal and temporal lobe networks, leading to long-term neurological and cognitive sequelae and refractory epilepsy (11); it is therefore possible that prompt use of an aggressive therapeutic approach helped, in this patient, to substantially modify both his short- and long-term disease course.

In conclusion, our patient presented a clinical picture lying between FIRES and autoimmune encephalitis. This case strengthens the view that FIRES might constitute the initial clinical presentation of a CNS inflammatory disease that could have, among multiple distinct etiologies, an autoimmune cause. It follows that immunological and specific second- or third-level investigations should be included in the diagnostic work up of patients with FIRES-like phenotypes and that PE can be an effective treatment in this subset of patients.

## Data Availability Statement

The original contributions presented in the study are included in the article/supplementary material, further inquiries can be directed to the corresponding author/s.

## Ethics Statement

Written informed consent was obtained from the minor(s)’ legal guardian/next of kin for the publication of any potentially identifiable images or data included in this article.

## Author Contributions

SMB, SO, SF, GI, MG, and DF participated in the patient’s care. MB and SMB contributed to the conception and design of the report. MB performed the literature search, prepared the figures, and drafted the manuscript. GI, MG, and DF wrote sections of the manuscript and prepared the figures. SMB and PV supervised the draft manuscript. All authors contributed to the revision of the manuscript, and read and approved the submitted version.

## Conflict of Interest

The authors declare that the research was conducted in the absence of any commercial or financial relationships that could be construed as a potential conflict of interest.

## Publisher’s Note

All claims expressed in this article are solely those of the authors and do not necessarily represent those of their affiliated organizations, or those of the publisher, the editors and the reviewers. Any product that may be evaluated in this article, or claim that may be made by its manufacturer, is not guaranteed or endorsed by the publisher.
